# Histological type and grade of breast cancer tumors by parity, age at birth, and time since birth: a register-based study in Norway

**DOI:** 10.1186/1471-2407-10-226

**Published:** 2010-05-21

**Authors:** Grethe Albrektsen, Ivar Heuch, Steinar Ø Thoresen

**Affiliations:** 1 Department of Public Health and Primary Health Care, University of Bergen, Bergen, Norway; 2 Department of Mathematics, University of Bergen, Bergen, Norway; 3 Gade Institute, University of Bergen, Bergen, Norway

## Abstract

**Background:**

Some studies have indicated that reproductive factors affect the risk of histological types of breast cancer differently. The long-term protective effect of a childbirth is preceded by a short-term adverse effect. Few studies have examined whether tumors diagnosed shortly after birth have specific histological characteristics.

**Methods:**

In the present register-based study, comprising information for 22,867 Norwegian breast cancer cases (20-74 years), we examined whether histological type (9 categories) and grade of tumor (2 combined categories) differed by parity or age at first birth. Associations with time since birth were evaluated among 9709 women diagnosed before age 50 years. Chi-square tests were applied for comparing proportions, whereas odds ratios (each histological type vs. ductal, or grade 3-4 vs. grade 1-2) were estimated in polytomous and binary logistic regression analyses.

**Results:**

Ductal tumors, the most common histological type, accounted for 81.4% of all cases, followed by lobular tumors (6.3%) and unspecified carcinomas (5.5%). Other subtypes accounted for 0.4%-1.5% of the cases each. For all histological types, the proportions differed significantly by age at diagnoses. The proportion of mucinous and tubular tumors decreased with increasing parity, whereas Paget disease and medullary tumors were most common in women of high parity. An increasing trend with increasing age at first birth was most pronounced for lobular tumors and unspecified carcinomas; an association in the opposite direction was seen in relation to medullary and tubular tumors. In age-adjusted analyses, only the proportions of unspecified carcinomas and lobular tumors decreased significantly with increasing time since first and last birth. However, ductal tumors, and malignant sarcomas, mainly phyllodes tumors, seemed to occur at higher frequency in women diagnosed <2 years after first childbirth. The proportions of medullary tumors and Paget disease were particularly high among women diagnosed 2-5 years after last birth. The high proportion of poorly differentiated tumors in women with a recent childbirth was partly explained by young age.

**Conclusion:**

Our results support previous observations that reproductive factors affect the risk of histological types of breast cancer differently. Sarcomas, medullary tumors, and possible also Paget disease, may be particularly susceptible to pregnancy-related exposure.

## Background

Some recent studies have indicated that reproductive factors, as well as other hormone-related risk factors, affect the risk of histological types of breast cancer differently [1-7], possibly reflecting a different etiology of disease according to histological type. Lobular tumors have shown an association with age at first birth which is stronger than for other histological types [1-3,7]. Moreover, increasing parity may be associated with an increased risk of medullary tumors [3,7], in contrast to the general protective effect.

The long-term protective effect of a childbirth on breast cancer risk is preceded by a short term adverse effect [8,9]. The transient increase in risk reaches a maximum about 5-7 years postpartum [8,9]. Breast cancer patients diagnosed during pregnancy and <2 years after birth often have a poor prognosis [10-20]; the tumors are often estrogen-receptor negative and at an advanced stage at time of diagnosis [10-14]. The proportion of late stage tumors have also been found to be high in women diagnosed 2-5 years after birth [21]. Hormone receptor status and other clinical characteristics of tumors, as well as prognosis, have been found to differ by histological type [22-27]. It is possible that a pregnancy triggers growth only of certain histological types of breast cancer tumors. Nevertheless, few studies have examined whether breast cancer tumors diagnosed in the first years after a childbirth tend to be of a particular histological type. Further knowledge about this issue, as well as about underlying biological mechanisms for the adverse effect of a pregnancy, may give valuable information for improved and more individualized treatment.

In the present register-based study, we examined associations between histological type and grade of breast cancer tumors and traditional reproductive risk factors, as well as time intervals since first and last (most recent) childbirth. Potential relations with age at diagnosis and family history of breast cancer were also explored.

## Methods

### Study population

Our study included information on 22,867 Norwegian female breast cancer patients diagnosed at ages 20-74 years (mean 50.8 years) during the period 1955 through 1999. Information on reproductive history, that is date of birth for a woman and all her children, was obtained from the Norwegian Population Registry. Information on reproductive history was regarded as complete for women born in 1925 and later. Since 1953, all cancer diagnoses in the country are by law reported to the Cancer Registry of Norway. Linkage of data from different national registers was possible using the unique personal identification number. The data file that served as basis for the present study was generated as part of a large, population-based prospective study on reproductive factors and cancer risk [9]. Information on presence or absence of family history of breast cancer, in terms of a breast cancer diagnosis in mother or sisters, was available for a subsample [28], including 7331 breast cancer cases in the present study. The study has been approved by the Norwegian Data Inspectorate and the Regional Committee for Medical and Health Research Ethics.

### Histological type and grade of tumor

Only cases classified as primary invasive malignant neoplasm of the breast (ICD 7^th ^revision, code 170) were considered. For diagnoses made before 1993, a rather crude classification of histological type was made. The classification system then changed from the 4-digit MoTNaC (histological code) to the 6-digit ICD-O-2 (morphological code). Among 23,039 breast cancer diagnoses in our cohort, histological or morphological code was recorded for 22,867 cases (99.3%). Certain histological codes were combined due to changes in classification system over time, and partly due to the low number of cases of each code. A total of 9 groups were defined for histological type (Table 1); ductal (n = 18,616), lobular (n = 1451), mucinous (n = 348), tubular (n = 257), medullary (n = 251), unspecified adenocarcinomas or carcinomas (n = 1250), Paget disease of the nipple (n = 332, most with an underlying ductal tumor), sarcomas (n = 96; 68 phyllodes tumors and 28 other/unspecified sarcomas) and 'other', that is all remaining valid histological types (n = 266, most common were mixed, tubulo-ductal, adenoid, papillary, apocrine).

**Table 1:** Histological characteristics.

	Abbr	Year of diagnosis		Total (%)
		1955 - 92	1993 - 99	
**Histological type**^a^				
1. Ductal (8500)^b,c^	Duct	9406 (75.6)	8046 (77.3)	18616 (81.4)
2. Lobular (8520,752:0-5)	Lob	526 ( 4.2)	925 ( 8.9)	1451 ( 6.3)
3. Mucinous (8480,8481)	Muc	146 ( 1.2)	202 ( 1.9)	348 ( 1.5)
4. Tubular (8211)	Tub	87 ( 0.7)	170 ( 1.6)	257 ( 1.1)
5. Medullary (8510,8512,7591)	Med	170 ( 1.4)	81 ( 0.8)	251 ( 1.1)
6. Other specified types	Other	58 ( 0.5)	208 ( 2.0)	266 ( 1.2)
7. Adenocarinomas/carcinomas, unspec. (8140, 8010, 8020)^d^	Unsp	641 ( 5.1)	609 ( 6.2)	1250 ( 5.5)
8. Paget disease, malign (854:0-3,752:6:8)	Paget	188 ( 1.5)	144 ( 1.4)	332 ( 1.5)
9. Sarcomas (9020,8800-8930,9120)^e^	Sarc	70 ( 0.2)	26 ( 0.3)	96 ( 0.4)
**Histological grade**				
Well/moderate (grade 1-2)		804 ( 6.5)	5256 (50.5)	6060 (26.5)
Poorly/undiff. (grade 3-4)		1104 ( 8.9)	2379 (22.9)	3484 (15.2)
Unknown/missing		10548 (84.7)	2776 (26.7)	13323 (58.3)

Histological types and grade of breast cancer tumors among 22,867 women aged 20-74 years

^a ^Four first digits in ICD-0-2 morphological code are given in parenthesis

^b ^From diagnoses before 1993: also including 7258 adenocarcinomas, morphological code 8143, and 1493 adenocarcinomas, code 814x, and 1164 carcinomas, code 8013

^c ^From diagnoses in 1993 and later: Also including 131 comedo cases, code 8501, and 63 ductal-lobular cases, code 8522

^d ^Including 343 poorly differentiated carcinomas, code 8020, and 397 cases classified as simplex carcinomas, code 823x before 1993

^e ^Including 68 phyllodes tumors

Histological grading (WHO classification system) was performed only for about 15% of cases diagnosed before 1993, but almost 75% of cases diagnosed in 1993 and later had histological grade recorded (Table 1). A total of 9544 (41.7%) breast cancer cases in the present study had information on histological grade.

### Statistical analyses

Chi-square tests were applied to examine whether the proportions of histological types (9 categories) or histological grade (2 combined categories, grade 1-2; well/moderately differentiated, and grade 3-4; poorly/undifferentiated tumors) differed by age at diagnoses, family history of breast cancer, or reproductive factors. To evaluate which categories had largest impact on the overall test for association, and in what direction, we calculated the cell specific contribution to the chi-square statistic (without squaring). An absolute value of this quantity equal to or above 1.96 corresponds roughly to an independent significant contribution at the 5% level. The corresponding critical value for a nominal significance level of 10% is 1.64. A negative value indicates fewer cases, whereas a positive value indicates more cases than expected by chance.

Polytomous logistic regression analysis was applied to examine whether associations between the exposure variables and any specific histological type of breast cancer differed from that seen with ductal tumors, the most common histological type. Odds-ratios (OR) for linear trend through ordered categories of the exposure variables were estimated. An OR-value of 1 indicates that the exposure variable considered is equally associated with the histological types that are compared (each type vs. ductal), indicating a similar risk pattern. If the exposure variable is positively associated with risk, an OR-value >1 indicates a stronger association, whereas an OR-value <1 indicates a less pronounced association than for ductal carcinomas. Binary logistic regression analyses were applied in the analyses of association with histological grade. The OR for observing a poorly differentiated tumor (grade 3-4) rather than a well/moderately differentiated tumor (grade 1-2), with increasing levels of an exposure variable (linear trend), was then estimated.

The analyses of time interval since a childbirth were restricted to women diagnosed before age 50 years (9709 and 3209 cases with complete information for histological type and grade, respectively). Diagnoses during pregnancy (DP), defined as starting 9 months prior to date of birth, and diagnoses before 2 years after delivery, comprised a separate category, whereas subsequent time categories were in 4 or 5 year intervals. The analyses with time interval since birth as main exposure variable were adjusted for age at diagnosis and parity, whereas the analyses of parity and age at first birth were adjusted for age at diagnosis and mutually adjusted for each other.

## Results

### Histological type of tumor by age at diagnosis and family history

Histological types of breast cancer tumors differed significantly by age at diagnosis (Table 2). The proportion of ductal carcinomas, the most common type (81.4%), was rather constant across age groups, but tended to be slightly less common in women diagnosed below age 30 years and above age 60 years. The proportion of lobular tumors, accounting for 6.3% of all cases, increased markedly with increasing age. Increasing trends were seen also for mucinous tumors (1.5%), tubular tumors (1.1%), and Paget disease (1.5%). In contrast, unspecified carcinomas (5.5%), medullary tumors (1.1%) and sarcomas (0.4%, mainly phyllodes tumors) were most common among women diagnosed below 30 years, and the proportion of these histological types decreased with increasing age.

**Table 2:** Histological type by age and family history.

		Histological type (%)								
		
	No.	Duct	Lob	Muc	Tub	Med	Other	Unsp	Paget	Sarc
**Age, diagn.**										
20-29 yr	297	78.8	1.0^(**)^	1.0	0.0^(*)^	3.7**	1.0	11.1**	1.3	2.0**
30-39 yr	3047	82.6	3.0^(**)^	0.9^(**)^	0.5^(**)^	1.9**	0.7^(**)^	8.7**	1.1	0.7**
40-49 yr	7957	82.1	5.4^(**)^	1.5	1.0	1.1	0.9^(**)^	6.3**	1.2^(*)^	0.5
50-59 yr	6868	82.9	7.4**	1.2^(**)^	1.3	1.0	1.1	3.2^(**)^	1.6	0.3
60-69 yr	4132	78.0	8.7**	2.4**	1.4*	0.7^(**)^	2.0**	4.8^(*)^	1.9**	0.2^(*)^
70-74 yr	566	74.2	9.5**	3.4**	1.9*	0.0^(**)^	2.7**	5.7	2.3*	0.4
P-value^a^	<0.001									
OR, unadj.^b^		-	1.4**	1.3**	1.3**	0.70**	1.5**	0.79	1.2**	0.65**
**Family history^c^**										
No	6505	81.8	7.0	1.4	1.4	1.3	1.3	4.2	1.2	0.5
Yes	826	81.1	7.7	0.8	1.7	1.5	1.6	3.9	1.6	0.1
P-value^a^	0.51									
< 50 yr										
No	4621	83.5	5.6	1.3	1.1	1.5	1.0	4.2	1.1	0.6
Yes	607	80.6	6.3	1.0	2.3**	1.6	1.6	4.6	2.0*	0.0^(*)^
P-value^a^	0.034									
≥ 50 yr										
No	1884	77.8	10.4	1.5	1.9	0.7	1.9	4.3	1.4	0.3
Yes	219	82.6	11.9	0.5	0.0	0.9	1.4	1.8	0.5	0.5
P-value^a^	0.18									

Histological types of breast cancer tumors by age at diagnosis and family history

^a ^Chi-square test for difference in proportions between groups, with significant cell-specific contributions to the chi-square statistic at 5% level marked with ** (Χ ≥ 1.96), and significant contributions at 10% level marked with * (Χ ≥ 1.64); in brackets if the observed number is lower than expected by chance

^b ^OR (each type vs. ductal tumors) for linear trend through ordered categories of exposure variable, significant trend (p < 0.05) marked with **

^c ^Mother/sister with a breast cancer diagnosis, restricted to 7331 cases with complete information

A significant association between histological type and familial risk was seen, but only in women diagnosed before age 50 years (Table 2). Breast cancer patients with a mother or sister diagnosed with the disease had a significantly higher proportion of tubular tumors. Paget disease was slightly more common, whereas sarcomas tended to be less common, though significant at 10% level only. In patients above 50 years, women with a familial risk had in general either ductal tumors (82.6%) or lobular tumors (11.9%). Other subtypes occurred mainly in women with no familial risk.

### Histological type of tumor by parity and age at first birth

Histological type of breast cancer tumors differed significantly by parity and maternal age at first birth (Table 3). Mucinous tumors, and also unspecified adenocarcinomas/carcinomas, were significantly more common in nulliparous than parous women. Sarcomas, mainly phyllodes tumors, also appeared with a higher proportion in nulliparous women, though not significantly. The difference between nulliparous and parous women was significant only in women diagnosed before age 50 years (p = 0.0003).

**Table 3:** Histological type by parity and age at first birth.

		Histological type (%)								
		
	No.	Duct	Lob	Muc	Tub	Med	Other	Unsp	Paget	Sarc
**Parity**										
0	3251	80.4	5.8	2.0**	1.1	1.1	1.0	6.7**	1.3	0.6*
≥ 1	19616	81.6	6.4	1.4	1.1	1.1	1.2	5.3	1.5	0.4
P-value*	0.004									
***Parous***										
1	3493	81.3	5.8	2.0**	1.4	0.8	1.3	5.7	1.3	0.4
2	8624	81.5	6.9	1.3	1.1	1.2	1.1	5.2	1.3	0.4
3	5123	81.9	6.2	1.5	1.1	1.1	1.1	5.3	1.4	0.3
≥ 4	2376	81.6	6.3	1.1	0.8	1.3	1.4	4.7	2.4**	0.3
P-value ^a^	0.007									
OR, unadj.^b^		-	1.0	0.87**	0.88**	1.1	1.0	0.95**	1.2**	0.88
OR, adjust.^b^^,^^c^		-	1.0	0.84**	0.82**	1.1	1.0	1.0	1.2**	0.90
**Age at first birth (yr)^d^**										
<20	2199	83.0	5.1^(**)^	1.3	1.2	1.7**	1.0	4.7	1.4	0.5
20 - 24	8610	82.0	6.2	1.4	1.2	1.1	1.2	5.1	1.4	0.4
25 - 29	6021	80.9	6.5	1.5	1.1	0.9	1.2	5.9**	1.6	0.4**
≥ 30	2782	80.4	7.9**	1.6	0.9	1.0	1.3	5.0	1.5	0.4
P-value ^a^	0.007									
OR, unadj.^b^		-	1.1**	1.1	0.92	0.82**	1.1	1.1	1.2	0.97
OR, adjust.^b,c^		-	1.1**	0.98	0.84**	0.87**	1.1	1.1**	1.1	0.98

Histological types of breast cancer tumors by parity and maternal age at first birth

^a ^Chi-square test for difference in proportions between groups, with significant cell-specific contributions to the chi-square statistic at 5% level marked with ** (Χ ≥ 1.96), and significant contributions at 10% level marked with * (Χ ≥ 1.64); in brackets if the observed number is lower than expected by chance

^b ^OR (each type vs. ductal tumors) for linear trend through ordered categories of exposure variable, significant trend (p < 0.05) marked with **

^c ^Adjusted for age at diagnosis, age at first birth/parity

^d ^Parous women only; four women excluded due to invalid value for age at first birth

Among parous women, the proportion of mucinous tumors decreased with increasing parity; the trend estimates indicated an inverse association with risk stronger than that for ductal tumors (Table 3). A similar pattern was seen for tubular tumors. In contrast, the proportion of Paget disease was significantly greater in women of high parity, with a significant increasing trend when contrasting to ductal tumors, indicating a less pronounced protective effect of parity in relation to Paget disease. The proportion of medullary tumors also appeared to be highest among multiparous women, but no independent significant contribution to the overall test for association was seen.

The proportion of lobular tumors, and also unspecified carcinomas increased steadily with increasing age at first birth (Table 3). In contrast, an inverse association was seen with medullary and tubular tumors.

### Histological type of tumor by time interval since a childbirth

Among women diagnosed before age 50 years, a significant overall association between histological type and time interval since first, as well as last birth was observed (Table 4). The proportion of ductal tumors was particularly high in women diagnosed <2 years after first birth (86.7%, and 90.4% in analyses restricted to uniparous women), and 15-19 years after last birth (85.0%), but the numbers were not significantly higher than the expected counts for these categories. Lobular, tubular and medullary tumors, unspecified carcinomas and also sarcomas made independent significant contributions to the overall tests for association with time since birth (details given below).

**Table 4:** Histological type by time interval since birth.

		Histological type (%)								
		
	No.	Duct	Lob	Muc	Tub	Med	Other	Unsp	Paget	Sarc
**Years since first birth**^a^										
DP, < 2	83	86.7	1.2	0.0	0.0	2.4	0.0	7.2	0.0	2.4**
2-5	459	80.4	2.0^(**)^	1.1	0.2	1.1	1.1	11.8**	1.5	0.9
6-9	869	80.4	3.7	0.9	0.6	2.2**	1.0	9.1**	1.3	0.8
10-14	1789	81.6	4.8	1.0	0.5^(*)^	1.3	0.7	8.4**	1.3	0.4
15-19	2471	82.3	4.6	1.0	0.7	1.4	1.0	7.5	1.0	0.5
≥ 20	4038	83.7	5.3*	1.4	1.3**	1.1	0.8	4.7^(**)^	1.2	0.4
P-value ^b^	<0.001									
OR, unadj.^c^		-	1.1**	1.1	1.5**	0.89**	0.97	0.81**	0.97	0.81**
OR, adjust.^c,d^		-	0.91**	0.94	1.2	1.2**	0.94	0.84**	0.97	0.93
OR, adjust.^c,e^		-	0.93**	1.0	1.3	1.2	0.96	0.83**	0.94	0.93
**Years since last birth**^f^										
DP, < 2	456	81.1	1.5^(**)^	0.7	0.0^(**)^	1.1	0.2	12.5	0.9	2.0**
2-5	1518	80.3	3.8	0.9	0.4^(*)^	2.0**	0.9	9.6**	1.7*	0.3
6-9	1654	81.6	4.2	1.0	0.8	1.5	1.0	8.2**	1.4	0.4
10-14	2336	82.7	5.2	1.0	0.9	1.1	0.8	7.1**	0.8^(*)^	0.4
15-19	2166	85.0	5.0	1.3	0.8	1.1	0.8	4.3^(**)^	1.2	0.4
≥ 20	1282	82.2	5.7*	1.5	1.4**	1.4	1.1	4.5^(**)^	1.3	0.7
P-value ^b^	<0.001									
OR, unadj.^c^		-	1.1**	1.1	1.3	0.92**	1.1	0.80**	0.95	0.91
OR, adjust.^c,d^		-	0.95**	1.0	1.1	1.2**	1.1	0.81**	0.92	1.1
OR, adjust.^c,e^		-	0.92**	0.96	1.1	1.3**	1.1	0.79**	0.93	1.1

Histological types of breast cancer tumors by time interval since a childbirth

^a ^Results based on analyses of 9709 parous women diagnosed before age 50 years

^b ^Chi-square test for difference in proportions between groups, with significant cell-specific contributions to the chi-square statistic at 5% level marked with ** (Χ ≥ 1.96), and significant contributions at 10% level marked with * (Χ ≥ 1.64); in brackets if the observed number is lower than expected by chance

^c ^OR (each type vs. ductal tumors) for linear trend through ordered categories of exposure variable, significant trend (p < 0.05) marked with **

^d ^Adjusted for age at diagnosis

^e ^Adjusted for age at diagnosis and parity

^f ^Results based on analysis of 9412 of women diagnosed before age 50 years, with singleton births only (1^st ^to 5^th ^birth)

Lobular tumors accounted for 4.7% of all cases diagnosed below 50 years (third most common type). The crude proportion of lobular tumors increased markedly with increasing time interval since birth. However, results from the age-adjusted analyses showed an association in the opposite direction, indicating an inverse association with risk stronger than that for ductal tumors (Table 4). Additional adjustment for parity did not affect the trend estimate notably. Tubular tumors (0.9%) occurred with a significantly higher proportion in women with a long time interval since birth, but no significant trend was seen in the adjusted analysis (Table 4).

Medullary tumors, accounting for 1.3% of cases (fourth most common), occurred with a significantly higher proportion in women diagnosed 6-9 years after first birth, and 2-5 years after last birth. These time periods frequently overlap, since most women deliver their subsequent children within a few years. However, the age-adjusted trend estimate showed a significant increase rather than decrease in the proportion of medullary tumors with increasing time since birth (Table 4). The positive trend seen with time since last birth was slightly strengthened by additional adjustment for parity.

Unspecified adenocarcinomas/carcinomas (6.9%, second most common), occurred with a significantly higher proportion in women diagnosed <2 years after last birth. A significant decreasing trend was seen both with time interval since first and last birth (Table 4). Sarcomas, mainly phyllodes tumors, though rare (0.5%), occurred with a significantly higher frequency among women diagnosed <2 years after first birth, as well as <2 years after last birth. The initial significant decreasing trend with increasing time since birth was weakened and no longer significant in the adjusted analyses (Table 4).

Paget disease (1.2%) appeared to be more common in women diagnosed 2-5 years after last birth compared to any other time period since birth (Table 4). The number of cases was higher than the expected count for this category, but significant at 10% level only. The estimated decreasing trend with increasing time since birth did not reach statistical significance (Table 4).

### Histological grade of tumor by age at diagnosis and family history

The proportion of poorly differentiated tumors decreased steadily with increasing age (Table 5). This age trend was seen both in nulliparous and parous women, but was particularly pronounced for nulliparous women. The proportion of missing values was highest among the youngest women, both in nulliparous and parous women. Nevertheless, a decreasing trend with age was seen only among nulliparous women when including a missing category in the analysis (results not shown).

**Table 5:** Histological grade by age and family history.

	All women (n = 9544)		Nulliparous Women		Parous Women	
	
	Total no.	Grade 3-4 (%)	Total no.	Grade 3-4 (%)	Total no.	Grade 3-4 (%)
**Age at diagnosis**						
20-29	72	58.3**	25	68.0**	47	53.2*
30-39	818	54.8**	135	57.0**	683	54.3**
40-49	2793	43.2**	317	47.3**	2476	42.7**
50-59	3006	33.4^(**)^	371	34.5	2635	33.2^(**)^
60-69	2429	28.6^(**)^	343	28.3^(**)^	2086	28.6^(**)^
70-74	426	20.7^(**)^	70	18.6^(**)^	356	21.1^(**)^
P-value^a^		<0.001		<0.001		<0.001
OR, unadjusted^b^		0.70**		0.65**		0.71**
**Family history**^c^						
No	3363	36.9	536	36.9	2827	36.9
Yes	406	38.9	76	48.7	330	36.7
P-value^a^		0.43		0.049		0.94
< 50 yr						
No	2221	40.8	307	45.0	1914	40.2
Yes	269	44.2	43	62.8	226	40.7
P-value^a^		0.28		0.028		0.88
≥ 50 yr						
No	1142	29.2	229	26.2	913	30.0
Yes	137	28.5	33	30.3	104	27.9
P-value^a^		0.85		0.62		0.65

Histological grade of breast cancer tumors by age at diagnosis and family history

^a ^Chi-square test for difference in proportions between groups, with significant cell-specific contributions to the chi-square statistic at 5% level marked with ** (Χ ≥ 1.96), and significant contributions at 10% level marked with * (Χ ≥ 1.64); in brackets if the observed number is lower than expected by chance

^b ^OR (grade 3-4 vs. grade 1-2) for linear trend through ordered categories of exposure variable, with significant test for trend (p < 0.05) marked with **

^c ^Mother/sister with a breast cancer diagnosis, restricted to 3769 cases with complete information

No significant overall association was found with family history of breast cancer (Table 5). Among nulliparous women, however, the proportion of patients with a poorly differentiated tumor was significantly higher among patients with a familial risk, though only in women diagnosed before the age of 50 years.

### Histological grade of tumor by parity and age at first birth

No overall significant difference in the proportion of high-grade tumors was seen between nulliparous and parous women (Table 6). Among parous women, the proportion of poorly differentiated tumors increased significantly with increasing parity, and decreased significantly with increasing age at first birth (Table 6). In analyses with mutual adjustment, only the trend with parity remained significant (Table 6). The proportion of missing values was rather constant across these groups, and the observed trends were not notably affected by inclusion of a missing category in the analyses (results not shown).

**Table 6:** Histological grade by parity and age at first birth.

	Total no.	Grade 3-4 (%)
**Parity**		
0	1261	38.2
≥ 1	8283	36.2
P-value ^a^		0.17
***Parous women:***		
1	1446	34.8
2	3767	35.7
3	2100	36.4
≥ 4	970	40.1**
P-value ^a^		0.045
OR, unadjusted^b^		1.1**
OR, adjusted^b^^**,**^^c,d^		1.1**
**Age at first birth (yr)**^d^		
<20 yr	1008	37.7
20 - 24 yr	3672	37.7
25 - 29 yr	2491	35.0
≥ 30 yr	1110	32.7^(**)^
P-value ^a^		0.0076
OR, unadjusted^b^		0.92**
OR, adjusted^b,c^		0.98

Histological grade of breast cancer tumors by parity and maternal age at first birth

^a ^Chi-square test for difference in proportions between groups, with significant cell-specific contributions to the chi-square statistic at 5% level marked with ** (Χ ≥ 1.96), and significant contributions at 10% level marked with * (Χ ≥ 1.64); in brackets if the observed number is lower than expected by chance

^b ^OR (grade 3-4 vs. grade 1-2) for linear trend through ordered categories of exposure variable, with significant test for trend (p < 0.05) marked with **

^c ^Adjusted for age at diagnosis and age at first birth/parity

^d ^Two cases excluded due to missing value for age at first birth

### Histological grade of tumor by time interval since a childbirth

Among women diagnosed before age 50 years, the proportion of poorly differentiated tumors decreased steadily with increasing time since first and last birth (Table 7). The association appeared to be particularly pronounced in relation to time interval since last birth; more than 60% of the tumors diagnosed within 2 years after most recent birth were poorly differentiated tumors. Despite some variation in the proportion of missing values across groups, the observed associations were not notably affected by inclusion of a missing category in the analyses. In the age-adjusted analyses, however, no significant association was seen with time since first birth, and the association with time since last birth was weakened, but on the border of statistical significance (p = 0.052). Additional adjustment for parity, however, weakened the association (Table 7).

**Table 7:** Histological grade by time interval since birth.

	**First birth**^a^		**Last birth**^b^	
	
	Total no.	Grade 3-4 (%)	Total no.	Grade 3-4 (%)
**Years since birth**				
DP, <2	31	54.8	132	62.1**
2 - 5	133	52.6	352	52.3**
6 - 9	239	49.8	467	46.7
10 - 14	505	48.1	728	44.8
15 - 19	754	46.4	753	43.0
≥ 20	1547	42.5^(*)^	665	40.2^(*)^
P-value^c^		0.026		<0.001
OR, unadjusted^d^		0.90**		0.88**
OR, adjusted^d,e^		1.00		0.94
OR, adjusted^d,f^		0.98		0.98

Histological grade of breast cancer tumors by time interval since a childbirth

^a ^Results based on 3209 parous women diagnosed before age 50 years

^b ^Results based on 3097 parous women diagnosed before age 50 years, with singleton births only (1^st ^to 5^th ^birth)

^c ^Chi-square test for difference in proportions between groups, with significant cell-specific contributions to the chi-square statistic at 5% level marked with ** (Χ ≥ 1.96), and significant contributions at 10% level marked with * (Χ ≥ 1.64); in brackets if the observed number is lower than expected by chance

^d ^OR (grade 3-4 vs. grade 1-2) for linear trend through ordered categories of exposure variable, significant trend (p < 0.05) marked with **

^e ^Adjusted for age at diagnosis

^f ^Adjusted for age at diagnosis and parity

## Discussion

In this large register-based study of breast cancer cases, histological type and grade of tumors were found to differ significantly by age, parity, and maternal age at first birth. The difference between nulliparous and parous women by histological type was most pronounced among women at pre- and perimenopausal ages. Ductal tumors represented the most common histological type in all subgroups defined by time interval since birth. Nevertheless, certain histological types appeared to be more susceptible to an adverse effect of a pregnancy. Further knowledge on this issue may provide important information in the treatment of women diagnosed with breast cancer shortly after a childbirth.

The results from this study need to be interpreted with some caution. Our study comprised information on breast cancer cases diagnosed in Norway in the period 1955-99. The histological classification system and procedures have varied over time, and may also have varied at the individual as well as institutional level. Still, the distribution of histological types across the two main calendar periods considered (before/after 1993), was roughly the same. Moreover, the observed age distribution of histological types appeared to be rather similar to that reported by others [23,29], except for a slightly higher proportion of ductal tumors. Histological grading of breast cancer tumors was not commonly carried out in Norway before 1993. Although the missing rate was high, there was no reason to believe that the information on histological grade was not missing at random. In some cases, however, the missing rate differed between the groups that were compared, making interpretation of results more difficult. Nevertheless, it is unlikely that our results can be completely explained by misclassification, or incompleteness of data.

Established associations between reproductive factors and the risk of breast cancer will mostly reflect associations with ductal tumors, which comprise about 80% of all breast cancer cases. A complete risk analysis of each subtype, in particular when considering time-related effects of a pregnancy, is difficult due to the low number of cases of certain histological types. However, the population at risk for each histological type in our cohort is the same, and information from the present study with respect to contrasting histological types, is thus comparable to the few previous studies that have calculated risk estimates specific to histological type, despite different measures of effect. Most of these studies, however, have focused on traditional reproductive factors, rather than time-related effects of a childbirth.

### Histological type of tumor by parity and age at first birth

Consistent with results from previous studies [1,7], our results indicated that the overall protective effect of a childbirth in relation to breast cancer risk may be particularly pronounced for mucinous tumors. In our study, a similar pattern was seen for tubular tumors. Our results also give some support to previous findings [3,7] of a less pronounced protective effect of parity in relation to medullary tumors, perhaps even an association in the opposite direction. In our study, Paget disease also appeared to be more common in women of high parity. Few previous studies have considered this disease.

Results from some previous studies have indicated that lobular tumors may be more strongly associated with age at first birth than other histological types of breast cancer [1-3,7]. Our result is consistent with these findings. Tubular and medullary tumors, however, were less strongly associated with maternal age at first birth, as compared to ductal tumors.

### Histological type of tumor by time interval since a childbirth

The transient increase in risk of breast cancer shortly after a childbirth is assumed to be related to a growth enhancing effect of pregnancy-related factors on pre-malignant breast cells [8,21]. Biological features of malignant or pre-malignant breast cells may affect susceptibility to pregnancy-related exposure and progression of disease into a clinically detectable phase [26,30,31]. A main objective of the present study was therefore to examine whether breast cancer tumors diagnosed shortly after a childbirth tend to be of a particular histological type. Women with a familial predisposition are more likely to have pre-cancerous lesions at a young age [32]. The number of cases in our study, however, was too low to perform an analysis separately for this subgroup. Nevertheless, results from overall associations between histological type and familial risk are briefly discussed.

The proportion of ductal tumors seemed to be particularly high among women diagnosed <2 year after first childbirth, but no clear trend with time since birth was seen. In one previous study of breast cancer diagnosed during pregnancy [19], all tumors were ductal carcinomas and 32 of 38 (84.2%) were poorly differentiated. Malignant sarcomas (mainly phyllodes tumor) occurred at significantly higher frequency in women diagnosed <2 years after birth. A rapid growth of a phyllodes tumor during pregnancy has been reported previously [33]. Patients with phyllodes tumors have in general been found to have a good prognosis [27,34], except those with large and poorly differentiated tumors [35,36].

The crude proportion of lobular tumors increased with increasing time since birth, but the age-adjusted trend estimate indicated an association in the opposite direction, with a decreasing trend in risk stronger than for ductal tumors. Lobular tumors have been found to be particularly sensitive to hormonal exposure [4,5], and may thus be susceptible to exposure to pregnancy hormones. On the other hand, such tumors tend to be more common among older women [3,37,38]. An excess familial risk has been noted [37-40], but was not confirmed in the present study.

Medullary tumors occurred with significantly higher frequency 2-5 years after last birth in our study. The unadjusted trend estimate indicated a decrease in the proportions of medullary tumors with increasing time since birth. The age-adjusted analyses, however, suggested an association in the opposite direction. The observed non-linear pattern with time since birth, with a peak 2-5 years after last birth, made interpretation of estimated linear trends somewhat difficult though. Thus, our data may indicate that medullary tumors are particularly susceptible to pregnancy-related exposure. Medullary tumors have previously been noted to progress rapidly from a pre-clinical to a clinically detectable phase [26]. The tumors are often associated with negative prognostic markers [22,23,29,41], but a favorable prognosis has been observed in most studies [24-27,38,41-43]. Medullary tumors have previously been found to be associated with a familial and/or hereditary risk, though not consistently [3,38,39,42-44]. No association with familial risk was seen in the present study.

Results from the present study also suggested that a pregnancy may have particular impact on the development of Paget disease. As far as we know, no previous studies have reported that this disease may be more common in the first years following a childbirth. Consistent with previous suggestions [45,46], most women diagnosed with Paget disease in the present study had an underlying tumor of ductal type. The tumor is often high grade and estrogen and progesterone receptor negative [45,47], and has been found to be positive also for other molecular markers that are frequently associated with more aggressive tumor behavior [47]. A high degree of inflammatory infiltrate in tumor-surrounding tissue has also been noted [46]. It has previously been suggested that inflammation-like processes in connection with the rebuilding of breast tissue after pregnancy and lactation may play a role for growth and spread of breast cancer tumors [48,49]. In the present study, Paget disease appeared to be more common in women with a familial risk.

The proportion of unspecified carcinomas decreased significantly with increasing time since birth in our study, both in unadjusted and adjusted analyses. The rather high proportion of unspecified carcinomas in women diagnosed shortly after birth, however, may reflect difficulties with classification of the tumor. About half the tumors within this subgroup were poorly differentiated.

### Histological grade of tumor by reproductive factors

The present finding that younger women, and women with recent childbirths more often have poorly differentiated tumors, is consistent with results from previous studies [20,50]. Results from our analyses with mutual adjustment, however, indicated that the high proportion of poorly differentiated tumors in women with a recent childbirth may be related to their generally younger age. On the other hand, the borderline significant association with time since last birth in the age-adjusted analyses suggested an effect of pregnancy-related factors rather than a confounding effect of age. Additional adjustment for order of birth, however, weakened the association with time interval since most recent birth.

Consistent with results from two previous studies [20,51], we observed a larger proportion of poorly differentiated tumors among women with an early first birth. No clear trend with age at first birth remained in our age-adjusted analyses, however. Rather unexpectedly, we observed an increase in the proportion of high-grade tumors with increasing number of births among parous women. The trend estimate did not change after adjustment for age at diagnosis and maternal age at first birth. A similar pattern was observed in another study [20], but the positive though non-significant trend with parity in that study was mainly related to a low proportion of high-grade tumors in nulliparous women, in contrast to that seen in our study.

## Conclusions

Consistent with findings from previous studies that have calculated risk estimates specific to histological subgroups, our results indicate that the associations between reproductive factors and the risk of breast cancer differ by histological type of the tumor. The overall protective effect of a pregnancy seemed to be particularly pronounced in relation to mucinous and tubular tumors. Differentiation of breast cells after a full-term pregnancy, but also a long-term reduction in prolactin levels, have been suggested to explain the protective effect of a childbirth on the risk of breast cancer. It is difficult to draw a final conclusion whether any of these hypotheses are more likely to explain associations with the risk of developing specific histological types of breast cancer tumors.

Certain histological types occurred with a significantly higher proportion in women diagnosed shortly after a childbirth, indicating a higher susceptibility to an unfavorable effect of pregnancy-related factors. Both hormonal and non-hormonal mechanisms have been suggested to explain the short-term adverse effect of pregnancy on breast cancer risk. Results from our multivariate regression analysis indicated that lobular tumors, presumably the most hormone-sensitive tumor type, occurred with higher frequency than expected shortly after a childbirth. The number of such tumors during this time period was very low though, making results from the multivariate analyses very imprecise. In view of findings from previous studies, the significantly higher proportion of women diagnosed with Paget disease in the first years following a childbirth, gives support to the hypothesis that an inflammation process in connection with pregnancy and lactation may contribute to progression of breast cancer disease. The higher frequency of women with Paget disease with increasing number of births may reflect a higher susceptibility to an inflammation in multiparous women.

The observed higher proportion of sarcomas and medullary tumors among women with a recent childbirth may be related to young age of the women, since we cannot rule out the possibility of residual confounding. The higher frequency of medullary tumors among multiparous women, however, is consistent with an unfavorable effect of a pregnancy on this particular histological type of breast cancer. Additional studies with more detailed information on histological type of tumor are needed to further explore these issues. Improved knowledge may have implications both for prevention and treatment of breast cancer disease.

## Competing interests

The authors declare that they have no competing interests.

## Authors' contributions

GA initiated the study, conducted statistical analyses and prepared initial draft and revisions of manuscript. IH contributed on methodological issues and ST contributed on medical issues. All authors contributed to the scientific content of the manuscript, commented on revisions and read and approved the final version.

## Pre-publication history

The pre-publication history for this paper can be accessed here:

http://www.biomedcentral.com/1471-2407/10/226/prepub
